# eHealth literacy and health responsibility among older adults: the serial indirect roles of self-efficacy and social support based on the health promotion model

**DOI:** 10.3389/fpubh.2026.1857565

**Published:** 2026-07-13

**Authors:** Yawen Wang, Guoxin Zhu, Xiaolong Ma, Hui Li, Yan Wang

**Affiliations:** 1 School of Medicine Management, Shandong First Medical University, Tai’an, Shandong Province, China; 2 School of Clinical and Basic Medical Sciences, Shandong First Medical University, Jinan, Shandong Province, China; 3 Shandong Port Medical Care and Health Management Group Co., Ltd., Qingdao, China; 4 College of Economics and Management, Shandong Agricultural University, Tai’an, China

**Keywords:** eHealth literacy, health responsibility, older adults, self-efficacy, social support

## Abstract

**Introduction:**

Guided by the Health Promotion Model and informed by Social Cognitive Theory, this cross-sectional study examined the association between eHealth literacy and health responsibility among older adults and explored whether self-efficacy and social support were indirectly involved in this association in a serial pattern.

**Methods:**

A convenience sample of 865 adults aged 60 years and older in Shandong Province, China, was surveyed using the eHealth Literacy Scale, the General Self-Efficacy Scale, the Social Support Rating Scale, and the health responsibility subscale of the Health-Promoting Lifestyle Profile II. Data were analyzed using SPSS 27.0 and the PROCESS macro.

**Results:**

eHealth literacy was positively associated with health responsibility and was also positively associated with self-efficacy and social support. The analysis further showed significant indirect associations involving self-efficacy alone, social support alone, and the serial combination of self-efficacy and social support in the association between eHealth literacy and health responsibility.

**Discussion:**

Higher eHealth literacy co-occurred with greater health responsibility, stronger self-efficacy, and greater social support in this cross-sectional sample of older adults. The findings are consistent with a theory-informed pattern linking eHealth literacy, self-efficacy, social support, and health responsibility. These results may inform future longitudinal research and health promotion efforts targeting older adults.

## 1 Introduction

The public health systems and healthcare services are under mounting pressure amid accelerating population aging, particularly in countries with rapidly growing older populations such as China (1). It is widely acknowledged that health-promoting behaviors are key factors of health status (2–4). Health-promoting behaviors are defined as self-initiated, multidimensional actions and thought patterns individuals adopt to maintain or improve their health, realize their potential, and increase life satisfaction (5–7). Previous studies suggest that these behaviors are influenced not only by eHealth literacy but also by factors such as age, perceived benefits, health beliefs, cultural adaptability, self-efficacy, and social support (8–12). Health responsibility refers to the extent to which individuals actively take responsibility for health management, which is typically reflected in health monitoring, preventive practices, and information seeking. According to China’s “2024 National Report on the Development of the Aging Cause,” the population aged 60 years and above reached 310 million by the end of 2024, representing the largest older population globally. This rapid demographic shift has created big challenges for healthcare and service systems for older adults (13–15). At the same time, the widespread adoption of digital medical technologies is reshaping how older adults manage their health, bringing both opportunities and new challenges (16, 17). Therefore, examining factors associated with health responsibility among older adults, and exploring how these factors are interrelated in the context of digital health, may contribute to the public health literature on healthy aging and digital health promotion (18, 19).

### 1.1 The association between eHealth literacy and health responsibility

eHealth literacy describes the capacity of individuals to locate, understand, communicate, apply, and assess health-related content in a dynamic environment of information exchange (20, 21). As digital healthcare technologies are expanding, a growing number of older adults are turning to online health resources for information and support (22, 23). Therefore, eHealth literacy has become a crucial factor associated with the health of older adults (24, 25). According to the health promotion model (HPM) (26), the present study considered eHealth literacy as a behavior-related cognitive factor that may be linked to health-promoting behaviors. As a new type of health literacy in the digital age, eHealth literacy is relevant to older adults’ ability to access, understand, and use online health-related information. Health responsibility refers to the extent to which individuals actively assume responsibility for health management, specifically manifested in behaviors such as health monitoring, preventive practices, and information seeking (27). Previous studies have shown that eHealth literacy is positively associated with health-promoting behaviors, and has also been linked to health responsibility and overall health status (28–30). However, current research mainly focuses on college students (31), nurses (28), and older adults with chronic diseases (32), while research examining eHealth literacy in relation to health responsibility in the general older adult population is relatively lacking. Therefore, we propose Hypothesis 1 (H1).

*H1*: eHealth literacy is positively associated with health responsibility among older adults.

### 1.2 The indirect role of self-efficacy in the association between eHealth literacy and health responsibility

Self-efficacy is described as an individual’s belief in their ability to achieve specific goals in a particular domain (33, 34), and is influenced by factors such as learning, feedback, and experience (35). The Health Promotion Model (HPM) highlights self-efficacy as a crucial factor in health promotion and connects it to health-related quality of life (36–38). Within this framework, self-efficacy is regarded as a factor that may be associated with individuals’ engagement in health management. Higher eHealth literacy may be associated with greater confidence in older adults in their ability to manage health-related tasks. Stronger self-efficacy may, in turn, be associated with more proactive health-management behaviors. Prior studies have also reported that self-efficacy may play an indirect role in the association between health literacy and health-promoting behaviors (12, 39). Based on this, we propose Hypothesis 2 (H2).

*H2*: Self-efficacy plays an indirect role in the association between eHealth literacy and health responsibility among older adults.

### 1.3 The indirect role of social support in the association between eHealth literacy and health responsibility

Social support refers to assistance available through an individual’s social network, including instrumental, emotional and informational resources, and it reflects the closeness of their connections with others (40). Because individuals live within and are shaped by their social environment, social support and interpersonal connections are often viewed as fundamental components of human function (41). The Health Promotion Model (HPM) identifies interpersonal influence as an important external factor relevant to health-promoting behaviors, and social support can be understood as a key component of interpersonal influence. This model suggests that support from family, friends, and healthcare providers may be associated with health responsibility by providing information, emotional encouragement, and behavioral examples (26). Chinese society, in particular, emphasizes a relationship structure centered on the family and based on filial piety, and social support among older adults often comes primarily from children and relatives (42). As Social Cognitive Theory (SCT) suggests, health-related behaviors are shaped by the interaction between internal cognition and the external environment (43, 44). Previous research has suggested that social support may play an indirect role in the association between eHealth literacy and healthcare-seeking behavior in older adults with chronic diseases (45). However, limited research has examined whether social support is indirectly involved in the association between eHealth literacy and health responsibility, particularly among older adults in China. Based on this, we propose Hypothesis 3 (H3).

*H3*: Social support plays an indirect role in the association between eHealth literacy and health responsibility among older adults.

### 1.4 The serial indirect roles of self-efficacy and social support in the association between eHealth literacy and health responsibility among older adults

eHealth literacy, self-efficacy, and social support have all been linked to health-promoting behaviors and may also be relevant to health responsibility among older adults. The Health Promotion Model (HPM) provides a conceptual framework for understanding how cognitive, interpersonal, and environmental factors may be jointly related to health-promoting behaviors. Within this framework, eHealth literacy, self-efficacy, and social support can be viewed as conceptually related factors relevant to health responsibility (9, 26). According to this model, self-efficacy may be relevant to how individuals engage in health responsibility and to how they perceive or make use of social support.

Persons with higher self-efficacy may be more likely to actively seek support resources and can more effectively utilize the support received to fulfill health management responsibilities (46). Based on this theoretical consideration, the present study specified self-efficacy before social support in the hypothesized serial pathway. This ordering was based on theoretical reasoning rather than an assumption of temporal causality. According to Social Cognitive Theory, individuals with stronger perceived capability are more likely to actively seek, interpret, and use environmental resources. In the digital health context, older adults with higher eHealth literacy may develop greater confidence in managing health-related information and digital services. This confidence may further enable them to express health needs, seek assistance from family members or healthcare providers, and make more effective use of available support. Thus, eHealth literacy may be associated with higher self-efficacy, which in turn may be linked to the acquisition and utilization of social support; together, these factors may show a serial pattern of association with health responsibility among older adults. Prior research has identified a connection between social support and self-efficacy in adolescents (47). Among community-dwelling older adults, previous studies have suggested that eHealth literacy may be associated with health-related behaviors via self-efficacy and self-care abilities, but these studies were limited to community-dwelling older adults and did not fully explore the influence of external factors such as social support (39). Many studies have also found that self-efficacy is negatively correlated with psychological distress and positively correlated with social support (46, 48). In the population of older adults with chronic diseases, self-efficacy and social support have been reported to show a serial indirect association between eHealth literacy and healthcare-seeking behavior (45), but this has not yet been extended to the general older adult population. Based on this, we propose Hypothesis 4 (H4).

*H4*: The association between eHealth literacy and health responsibility among older adults is indirectly linked to self-efficacy and social support in a serial pattern.

**Figure 1:**
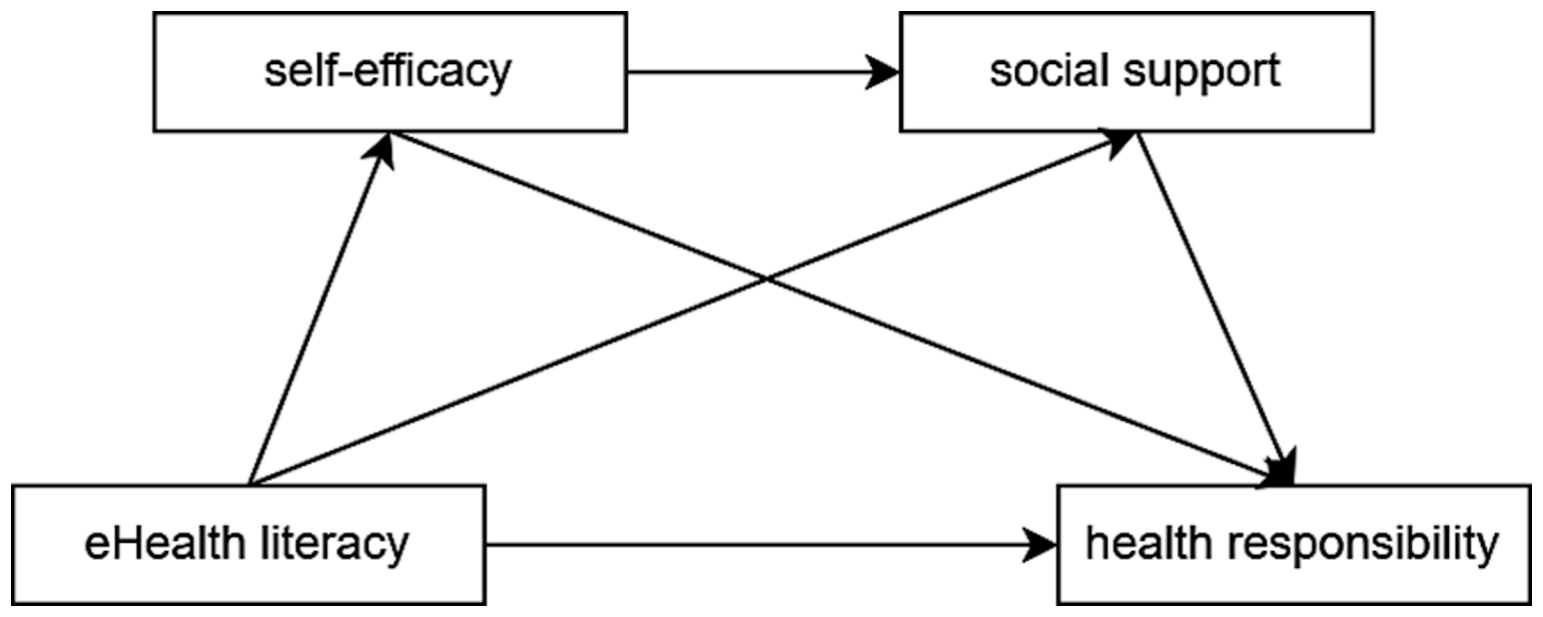
Hypothetical model.

Although some studies have explored the links between eHealth literacy and health behaviors, the existing literature has the following limitations. First, most studies focus on specific groups of individuals with chronic diseases, such as diabetes, heart disease, or chronic non-communicable diseases, lacking a systematic examination of the general older adult population. Second, although several studies have suggested indirect associations involving health-promoting behaviors or self-efficacy, limited research has examined whether eHealth literacy is indirectly linked to health responsibility through self-efficacy and social support in a serial pattern among older adults (39, 49, 50, 51). Third, existing studies mostly focus on the link between eHealth literacy and overall health-promoting behaviors or life qualities, with less attention paid to health responsibility as a core dimension (52). Guided by the Health Promotion Model and informed by Social Cognitive Theory, the present study examined whether self-efficacy and social support were indirectly involved in the association between eHealth literacy and health responsibility. This study may contribute evidence relevant to public health efforts aimed at promoting healthy aging and digital health engagement among older adults. The hypothetical model is illustrated in Figure 1.

## 2 Materials and methods

### 2.1 Participants

This study was conducted from August to September 2025, surveying 921 older adults in 16 cities of Shandong Province, China, using convenience sampling. Participants were recruited through community health service centers and senior activity centers, and the survey was conducted using online questionnaires. Inclusion criteria for the questionnaire were: (1) provision of informed consent and voluntary participation in the study; (2) age ≥ 60 years; (3) basic literacy skills. The exclusion criteria for the questionnaire were: (1) completion time less than 3 min; (2) filling of questionnaire with a single answer; (3) cognitive impairment, severe visual or hearing impairment, inability to complete the survey, or recent major health crisis (e.g., hospitalization). In total, 921 questionnaires were obtained. After removing 56 invalid samples, the actual number of valid questionnaires was 865, resulting in a final valid questionnaire rate of 93.92%. Statistics show that 322 respondents were aged 60–64 (37.2%), 161 were aged 65–69 (18.6%), 122 were aged 70–74 (14.1%), and 260 were aged ≥75 years (30.1%). A total of 865 older adults participated, including 456 men and 409 women. Among them, 466 lived in rural areas, while 399 lived in urban areas. Detailed demographic characteristics of the study sample are presented in Table 1. The study adhered to the ethical guidelines of the Declaration of Helsinki. All participants provided written informed consent and the study received the ethical approval from the Ethics Committee of Shandong First Medical University (approval No. R202508120459).

**Table 1:** Basic information (*n* = 865).

Variables	60–64 years	65–69 years	70–74 years	≥75 years	*χ* ^2^	*p*
Gender, *n* (%)					53.584	<0.001
Male	204 (63.4)	96 (59.6)	67 (54.9)	89 (34.2)		
Female	118 (36.6)	65 (40.4)	55 (45.1)	171 (65.8)		
Residence, *n* (%)					43.334	<0.001
Rural	202 (62.7)	100 (62.1)	67 (54.9)	97 (37.3)		
Urban	120 (37.3)	61 (37.9)	55 (45.1)	163 (62.7)		
Educational level, *n* (%)					139.151	<0.001
No formal schooling	134 (41.6)	30 (18.6)	28 (23.0)	17 (6.5)		
Primary school	62 (19.3)	38 (23.6)	28 (23.0)	98 (37.7)		
Junior high school	43 (13.4)	45 (28.0)	23 (18.9)	28 (10.8)		
High school/technical secondary school	43 (13.4)	20 (12.4)	17 (13.9)	45 (17.3)		
Junior college	25 (7.8)	19 (11.8)	17 (13.9)	40 (15.4)		
Bachelor’s degree or above	15 (4.7)	9 (5.6)	9 (7.4)	32 (12.3)		
Marital status, *n* (%)					52.961	<0.001
With spouse	204 (63.4)	118 (73.3)	64 (52.5)	105 (40.4)		
Without spouse	118 (36.6)	43 (26.7)	58 (47.5)	155 (59.6)		
Living arrangement, *n* (%)					77.340	<0.001
Living alone	139 (43.2)	36 (22.4)	39 (32.0)	37 (14.2)		
Living with spouse/partner/children	113 (35.1)	97 (60.2)	57 (46.7)	173 (66.5)		
Nursing home	70 (21.7)	28 (17.4)	26 (21.3)	50 (19.2)		

*χ*^2^ values were calculated using the chi-square test.

### 2.2 Demographic survey

Demographic data were gathered from all participants, including details such as age, gender, residence, marital status, educational level, and living arrangement.

### 2.3 Measurement tools

The questionnaire consisted of a brief sociodemographic section developed for this study and four previously validated instruments: the eHealth Literacy Scale (eHEALS), the General Self-Efficacy Scale (GSES), the Social Support Rating Scale (SSRS), and the health responsibility subscale of the Health-Promoting Lifestyle Profile II (HPLP-II).

#### 2.3.1 eHealth literacy scale (eHEALS)

The eHEALS was developed by Norman and Skinner (21) and consists of 8 items composed of three dimensions: application ability (AB), judgment ability (JA), and decision-making ability (DMB). A five-point Likert scale is used, ranging from “strongly disagree” to “strongly agree,” scored from 1 to 5 points, respectively. Total scores below 26 were considered indicative of lower eHealth literacy, whereas scores above this threshold reflected higher eHealth literacy. Previous researchers have reported the validity of this scale in older adults (53). In our study, the Chinese version revised by Guo Shuaijun et al. (54) was applied and the Cronbach’s *α* coefficient of eHEALS was 0.873, while the KMO value was 0.886. The eHEALS was used because it has been applied in older adults and validated in simplified Chinese versions, supporting its suitability for the present sample.

#### 2.3.2 General self-efficacy scale (GSES)

The GSES is a scale developed by German scholars Schwarzer et al. (55) to assess self-efficacy. This scale consists of 10 items, using a four-point Likert scale for scoring, ranging from “completely incorrect” to “completely correct,” scored from 1 to 4 points, respectively. The total score ranges from 10 (representing lower self-efficacy) to 40 (representing higher self-efficacy). This scale has been widely adopted and demonstrates good internal consistency (56). Our study used the Chinese version of the GSES revised by Wang Caikang et al. (57). In our study, the Cronbach’s *α* coefficient was 0.916 and the KMO value was 0.960. The GSES was used because perceived self-efficacy is a key cognitive construct in Social Cognitive Theory and health promotion research, and its Chinese version has been widely applied in adult and older adult populations.

#### 2.3.3 Social support rating scale (SSRS)

Social support was assessed using the Social Support Rating Scale (SSRS) created by Xiao Shuiyuan (58). The SSRS comprises 10 items, which are divided into three dimensions: objective support, subjective support, and utilization of support. It does not apply a uniform 1–4 scoring rule to all items. Items 1–4 and 8–10 are scored from 1 to 4, item 5 is calculated by summing support from family-member sources, and items 6 and 7 are scored according to the number of support sources selected. Therefore, the theoretical total score range of the SSRS in this study was 11–62, with higher scores indicating greater social support. Total score below 33 indicates low social support, 33–45 indicates average social support, and a score above 45 indicates high social support. This scale has been widely used in China (59). In our study, the Cronbach’s *α* coefficient of the SSRS was 0.827, and the KMO value was 0.890. The SSRS was used because it evaluates objective support, subjective support, and support utilization in the Chinese social context, making it suitable for assessing support resources among older Chinese adults.

#### 2.3.4 The health responsibility dimension of the health-promoting lifestyle profile II (HPLP-II)

The HPLP-II scale was originally developed by Walker et al. (60) based on the Health Promotion Model (HPM). The Chinese version revised by Cao Wenjun et al. comprises 40 items across 6 dimensions and has been widely adopted in China (61). Our study selected the health responsibility dimension from Cao Wenjun’s Chinese HPLP-II scale as the dependent variable. It includes health monitoring, preventive behaviors, and the pursuit of health-related information, which aligns well with the focus of eHealth literacy (62, 63). Furthermore, previous studies confirm its direct role in predicting health outcomes for older adults (64). The health responsibility dimension consists of 11 items rated on a four-point Likert scale (1 = “Never” to 4 = “Always”), where higher scores indicate greater health responsibility. In our study, the Cronbach’s *α* coefficient was 0.931 and the KMO value was 0.966.

### 2.4 Statistical methods

SPSS 27.0 was used for data processing and analysis. The online questionnaire platform required responses to all items before submission; therefore, no item-level missing values were present. Invalid questionnaires were excluded according to the criteria described above. Outliers were assessed using standardized z scores, with absolute z values greater than 3.00 considered indicative of outliers, and no cases exceeded this threshold. The normality of continuous variables was assessed using the Kolmogorov–Smirnov test, and the results indicated that the main continuous variables significantly deviated from a normal distribution (all *p* < 0.001). Therefore, associations between the variables were examined using Spearman’s correlation analysis. Differences in eHealth literacy, self-efficacy, social support, and health responsibility across age and educational-level groups were examined using the Kruskal-Wallis test. Cronbach’s *α* coefficients were used to assess the internal consistency of the scales, while exploratory factor analysis was conducted to evaluate the construct validity. In addition, Harman’s single-factor test was used to assess the potential impact of common method bias. Age, gender, residence, educational level, marital status, and living arrangement were included as covariates in all regression-based indirect association analyses. These variables were selected *a priori* because they are common sociodemographic factors related to older adults’ digital access, health literacy, social resources, and health-promoting behaviors. Model 6 in the PROCESS macro was applied to examine the serial indirect associations of self-efficacy and social support in the relationship between eHealth literacy and health responsibility. Given the cross-sectional design of this study, these analyses were used to estimate conditional associations along hypothesized pathways rather than causal mediation effects. The bootstrap method was employed for indirect associations testing, with the effect considered significant if the 95% confidence interval (CI) did not include zero. The two-tailed test was used, with a significance level of *α* = 0.05.

## 3 Results

### 3.1 Reliability analysis

The Cronbach’s α coefficients for the eHEALS, GSES, SSRS, and HPLP-II health responsibility subscale were 0.873, 0.916, 0.827, and 0.931, respectively, indicating good to excellent internal consistency (65).

### 3.2 Validity analysis

Exploratory factor analysis was conducted to examine the scales’ structure validity. The KMO and Bartlett’s tests showed that the KMO value was 0.967 and the Bartlett’s Test of Sphericity chi-square value was significant (*χ*^2^ = 20137.46, *p* < 0.001), suggesting that the data were suitable for factor analysis. The KMO values for the eHEALS, GSES, SSRS, and HPLP-II health responsibility subscale were 0.886, 0.960, 0.890, and 0.966, respectively.

### 3.3 Common method bias test

Because this study used a questionnaire survey and the data were based on self-reported responses from older adults, questionnaires were distributed and collected anonymously to reduce common method bias and improve the objectivity and reliability of the data. Harman’s single-factor test was used to assess common method bias. The results showed that 26 factors had eigenvalues greater than 1, and the first factor accounted for 38.923% of the total variance, which was below the critical threshold of 40%, indicating that common method bias was not a serious concern in this study.

### 3.4 Scores of participants’ eHealth literacy, self-efficacy, social support, and health responsibility

The mean total score for eHealth literacy was 24.51 ± 9.12, suggesting a relatively low level in this sample based on the scale cutoff reported above. The mean total score for social support was 37.96 ± 10.62, indicating an overall average level of social support. The mean item scores for self-efficacy (2.54 ± 0.91) and health responsibility (2.54 ± 0.90) suggested moderate levels overall. Detailed scores for each scale and dimension are presented in Table 2.

**Table 2:** Scores of eHealth literacy, self-efficacy, social support, and health responsibility (*n* = 865, M ± SD).

Variable	Number of items	Theoretical score range	Total score (M ± SD)	Item mean (M ± SD)
**eHealth literacy**	8	8 ~ 40	24.51 ± 9.12	3.06 ± 1.14
Application ability	5	5 ~ 25	15.28 ± 5.96	3.06 ± 1.19
Judgment Ability	2	2 ~ 10	6.11 ± 2.76	3.05 ± 1.38
Decision-making ability	1	1 ~ 5	3.11 ± 1.60	3.11 ± 1.60
**Self-efficacy**	10	10 ~ 40	25.37 ± 9.12	2.54 ± 0.91
**Social support**	10	11 ~ 62	37.96 ± 10.62	3.80 ± 1.06
Objective support	3	1 ~ 22	9.71 ± 2.64	3.24 ± 0.88
Subjective support	4	7 ~ 28	20.55 ± 7.16	5.14 ± 1.80
Utilization of support	3	3 ~ 12	7.69 ± 3.00	2.56 ± 1.00
**Health responsibility**	11	11 ~ 44	27.98 ± 9.86	2.54 ± 0.90

Bold rows indicate main scale scores, and indented rows indicate subscale or dimension scores.

Kruskal-Wallis tests were conducted to examine subgroup differences by age and educational level. Across age groups, significant differences were observed in self-efficacy (*H* = 12.816, *p* = 0.005), social support (H = 15.143, *p* = 0.002), and health responsibility (*H* = 19.898, *p* < 0.001), whereas no significant difference was found in eHealth literacy (*H* = 3.390, *p* = 0.335). Across educational-level groups, significant differences were observed in eHealth literacy (*H* = 11.253, *p* = 0.047), self-efficacy (*H* = 25.652, *p* < 0.001), social support (*H* = 20.305, *p* = 0.001), and health responsibility (*H* = 42.738, *p* < 0.001).

### 3.5 Correlation analysis

The results of the Spearman’s correlation analysis are detailed in Table 3. EHL was significantly positively associated with SE, SS, and HR. SE was also positively associated with SS and HR and SS was positively associated with HR. These results indicate that the main variables were significantly associated with one another, supporting further examination of indirect associations within the proposed theoretical framework.

**Table 3:** Correlation analysis among major variables.

Variable	EHL	SE	SS	HR
EHL	1			
SE	0.370**	1		
SS	0.396**	0.653**	1	
HR	0.509**	0.644**	0.675**	1

^**^p < 0.01; EHL, eHealth literacy; SE, self-efficacy; SS, social support; HR, health responsibility.

### 3.6 Serial indirect association model

**Figure 2:**
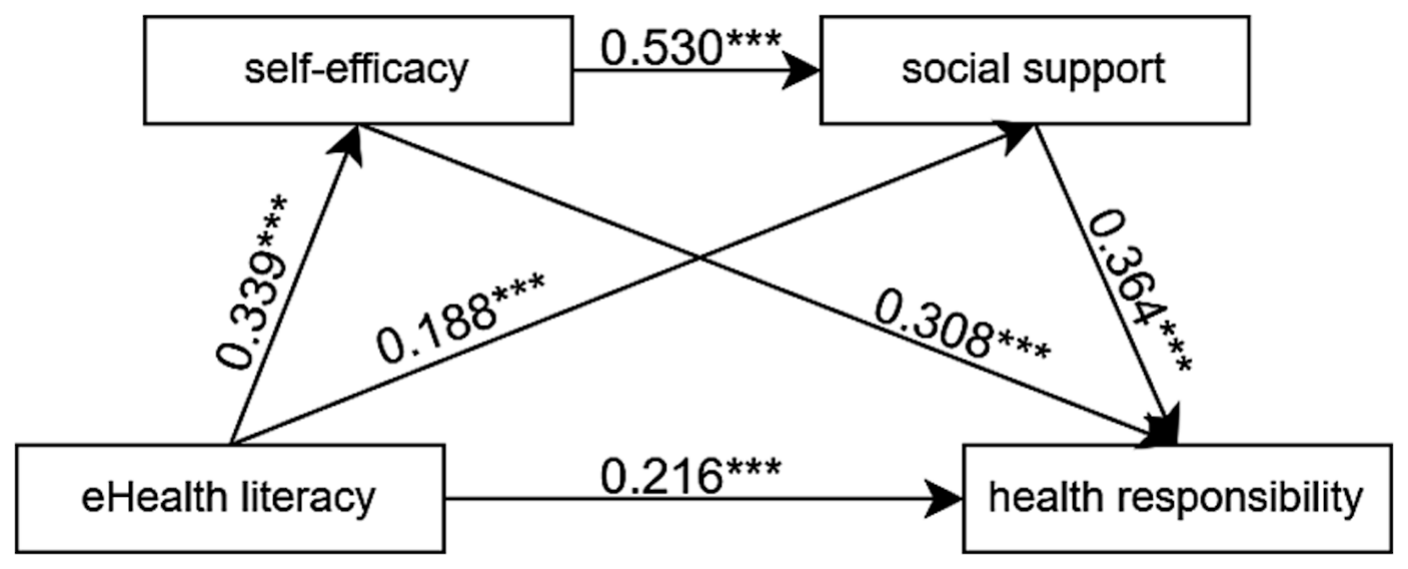
Serial indirect association model. Values are standardized path coefficients adjusted for age, gender, residence, educational level, marital status, and living arrangement. (^***^p < 0.001).

In this study, EHL and HR were specified as the focal variables, with SE and SS examined as variables involved in a serial indirect association pattern. PROCESS Model 6 estimated four separate regression equations: Model 1 estimated the total effect of EHL on HR; Model 2 regressed SE on EHL; Model 3 regressed SS on EHL and SE; and Model 4 regressed HR on EHL, SE, and SS. All models controlled for age, gender, residence, educational level, marital status, and living arrangement. The regression results showed that EHL significantly predicted SE, EHL and SE significantly predicted SS, and EHL, SE, and SS significantly predicted HR in the final model. These models explained 17.4% of the variance in SE, 44.2% of the variance in SS, and 62.2% of the variance in HR, respectively. Detailed regression results are presented in Table 4, and the adjusted standardized path coefficients are shown in Figure 2.

**Table 4:** Regression results from separate equations within PROCESS model 6 (*n* = 865).

Model	Dependent variable	Predictor	*B*	SE	*p*	95% CI	*β*
Model 1	HR	EHL	0.571	0.036	<0.001	0.501–0.642	0.453
Model 2	SE	EHL	0.372	0.035	<0.001	0.304–0.441	0.339
Model 3	SS	EHL	0.265	0.039	<0.001	0.188–0.342	0.188
	SS	SE	0.680	0.036	<0.001	0.609–0.750	0.530
Model 4	HR	EHL	0.272	0.030	<0.001	0.213–0.330	0.216
	HR	SE	0.353	0.032	<0.001	0.291–0.415	0.308
	HR	SS	0.325	0.025	<0.001	0.276–0.374	0.364

*B* represents unstandardized coefficients, and *β* represents standardized coefficients. All models were adjusted for age, gender, residence, educational level, marital status, and living arrangement. EHL, eHealth literacy; SE, self-efficacy; SS, social support; HR, health responsibility.

Bootstrap analysis with 5,000 resamples further showed that the standardized total effect of EHL on HR was 0.453, whereas the standardized direct effect was 0.216. The standardized total indirect effect was 0.238, accounting for 52.54% of the total effect. Specifically, three standardized indirect pathways were significant: EHL → SE → HR (effect = 0.104, 95% CI: 0.077 to 0.134), EHL → SS → HR (effect = 0.068, 95% CI: 0.046 to 0.092), and EHL → SE → SS → HR (effect = 0.065, 95% CI: 0.049 to 0.084). Because none of the bootstrap confidence intervals included zero, all indirect effects were statistically significant. Detailed standardized direct and indirect effects are presented in Table 5. Taken together, these findings supported H1, H2, H3, and H4 in terms of cross-sectional statistical associations. However, given the cross-sectional design, they should not be interpreted as evidence of temporal or causal mediation.

**Table 5:** Standardized direct and indirect pathways (*n* = 865).

Mechanism	Effect	BootSE	95% CI		Contribution rate (%)
			LLCI	ULCI	
Total effect	0.453	0.029	0.397	0.509	100
Direct effect	0.216	0.024	0.169	0.262	47.68
Total indirect effect	0.238	0.02	0.198	0.279	52.54
EHL → SE → HR	0.104	0.014	0.077	0.134	22.96
EHL → SS → HR	0.068	0.012	0.046	0.092	15.01
EHL → SE → SS → HR	0.065	0.009	0.049	0.084	14.35

Standardized effects are presented; 5,000 bootstrap samples; EHL, eHealth literacy; SE, self-efficacy; SS, social support; HR, health responsibility. The standardized coefficients in Table 4 and Figure 2 are based on ordinary least squares (OLS) regression using the full original sample, whereas the results in this table are derived from 5,000 bootstrap resamples. The minor difference of 0.001 between the two estimates reflects differences in computational logic, random sampling variation, and decimal rounding rules across software, which is a normal occurrence in mediation analysis.

Each contribution rate was calculated by dividing the individual pathway effect by the total effect. Because each value was rounded independently, the sum of contribution rates may not equal exactly 100%, which is a common statistical occurrence.

## 4 Discussion

### 4.1 Direct association between eHealth literacy and health responsibility

The study found a positive link between eHealth literacy and health responsibility among older adults, consistent with previous research by Xie and Mo (29), and Wang et al. (39) on older adults and Mousazadeh et al. (31) on university students. Based on the Health Promotion Model and Health Empowerment Theory, one possible interpretation is that older adults with higher eHealth literacy may have greater access to health information and resources and may therefore be more likely to report stronger health awareness and engagement in health-promoting behaviors (66, 67). These cross-sectional findings suggest that eHealth literacy may represent a relevant correlate of health responsibility, though the directionality and causal nature of this association require verification through longitudinal or experimental designs. While previous studies have predominantly examined the link between eHealth literacy and health-related behaviors among chronic disease patients (49), our study found that eHealth literacy remains significantly connected with health responsibility among older adults even in non-disease-specific contexts. This result expands the existing evidence and suggests that eHealth literacy may be an important consideration in public health efforts aimed at promoting healthy aging among older adults.

### 4.2 Discussion of indirect associations

#### 4.2.1 The indirect role of self-efficacy in the association between eHealth literacy and health responsibility

Our findings show that self-efficacy may be indirectly involved in the association between eHealth literacy and health responsibility. In the present cross-sectional sample, higher eHealth literacy co-occurred with stronger self-efficacy, which was in turn associated with greater health responsibility. While this pattern is consistent with an indirect association, causal inference is not warranted from this design. This pattern is consistent with prior studies by Wang et al. (39) and Park (68). According to Bandura’s social cognitive theory, self-efficacy reflects a person’s confidence in managing health information and engaging in health behaviors, which helps explain its positive link with health responsibility seen in our sample (43). In this context, higher self-efficacy may be associated with more positive health results in older adults. Overall, the study suggests that self-efficacy may represent an important correlate situated between eHealth literacy and health responsibility, a pathway that warrants testing in longitudinal research. Accordingly, future longitudinal and intervention studies could examine whether strengthening self-efficacy is relevant to promoting health responsibility among older adults (38).

#### 4.2.2 The indirect role of social support in the association between eHealth literacy and health responsibility

The results of the present analysis suggest that social support is indirectly involved in the association between eHealth literacy and health responsibility, alongside a remaining direct association. This finding is consistent with previous work by Xu et al., who reported that individuals with higher levels of eHealth literacy tend to report stronger social support and greater resilience (69). In our sample, social support was positively correlated with health responsibility among older adults, which may reflect the emotional support, access to health information, and behavioral guidance provided by social networks. This interpretation is consistent with the studies of Choi et al. (50). For example, strong interpersonal networks may help reduce psychological burden and technical barriers older adults face when seeking digital healthcare resources. These cross-sectional associations suggest that social support may be a relevant factor co-occurring with health responsibility, and that future longitudinal or intervention studies could further examine its relevance to health promotion among older adults.

A key point is that the social support model among older Chinese adults is deeply influenced by cultural norms, which may be important for understanding our findings. In particular, family dynamics, guided by the tradition of filial piety, often view children’s care and support for their parents not only as a moral duty but also as a manifestation of emotional bonds (70). This cultural background means that social support for Chinese older adults primarily comes from intergenerational networks within the family, rather than the peer support or community support models more common in Western societies (71). Specifically, children may help older adults use smartphones and health applications (instrumental support), explain and filter online health information (informational support), and encourage older adults to actively pay attention to their own health (emotional support) (72). The indirect role of social support found in our study may reflect this family centered support model. In China’s aging context, this finding suggests that the participation and support of family members, especially adult children, may be important in health promotion interventions for older adults, as they often help older adults use digital health resources, interpret online health information, and maintain confidence in health management.

#### 4.2.3 The serial indirect roles of self-efficacy and social support in the association between eHealth literacy and health responsibility

Our study identified a serial indirect association involving self-efficacy and social support in the association between eHealth literacy and health responsibility. This finding holds the following innovative significance.

From a theoretical perspective, our study may be among the first to examine this serial indirect association pattern in a general older adult population in China, thereby extending theory-informed research on health promotion in the context of digital health. Previous studies have mainly focused on the separate indirect roles of health-promoting behaviors or self-efficacy: Li et al. found that health-promoting behaviors were indirectly involved in the link between eHealth literacy and quality of life (51); Wang et al. confirmed that self-efficacy and self-care ability were linked to eHealth literacy and health-promoting behaviors in a serial indirect pattern (39); Choi also found that health self-efficacy was indirectly involved in the association between eHealth use and health-promoting behaviors (50). However, limited research has examined whether eHealth literacy is indirectly linked to health responsibility through the serial pattern of self-efficacy and social support. By examining this theory-informed serial pattern, the current study contributes to a clearer understanding of how eHealth literacy, self-efficacy, social support, and health responsibility may be interrelated in older adults.

Regarding the research environment, examining this serial pattern of associations in the Chinese context is significant, as existing literature mainly focuses on empirical studies on eHealth literacy and health behaviors in Western social contexts. However, notable differences exist between social support networks in Western and Chinese cultures (73). The Western model centers on individual independence, with older adults primarily relying on peer networks, community service systems, and professional institutions for support; while Chinese society is based on the family, and social support for older adults mainly comes from intergenerational interaction within the family (74). Our study identified a serial indirect association pattern in which eHealth literacy, self-efficacy, and social support were jointly associated with health responsibility. These cross-sectional findings are consistent with the theoretical sequence proposed by the Health Promotion Model, though longitudinal or experimental evidence is needed to establish temporal ordering and directionality. This result may reflect a unique pattern within the Chinese cultural context: older adults with higher self-efficacy may be better at expressing their health needs to their children and more willing to accept their children’s help, thus obtaining more family support and consequently exhibiting more proactive health responsibility behaviors (75). This finding suggests that health promotion models may also be informative in non-Western cultural contexts and may provide evidence for developing culturally appropriate health intervention strategies for older adults.

### 4.3 Limitations

This study has several limitations that should be acknowledged. First, the cross-sectional design does not permit causal inference or the establishment of temporal ordering among variables. The indirect associations reported here reflect cross-sectional associations consistent with the Health Promotion Model’s theoretical framework, and should not be interpreted as evidence of causal mediation. Longitudinal or experimental designs are needed to confirm directionality. Second, participants were recruited from Shandong Province using convenience sampling, which limits the generalizability of the findings beyond Shandong Province to broader Chinese and international older adult populations. In addition, because the survey was conducted online, older adults with limited digital access or difficulty completing online questionnaires may have been underrepresented. Therefore, the findings should be generalized cautiously beyond the sampled population. Third, this study employed only the health responsibility subscale of the HPLP-II rather than the full instrument. Whether the identified pathways extend to other dimensions of health-promoting behavior, such as nutrition, physical activity, or stress management, remains an open question for future research. Fourth, reliance on self-reported measures introduces the possibility of recall and social desirability bias, and the eHEALS captures perceived rather than objectively assessed digital health literacy.

## 5 Conclusion

Our study analyzed data from older adults in Shandong Province, China. The results demonstrated a positive cross-sectional association between eHealth literacy and health responsibility. Self-efficacy and social support were indirectly involved in this association in a theory-informed serial pattern. These findings are consistent with the theory-informed ordering proposed by the Health Promotion Model (HPM) in a Chinese older adult population, and may provide a foundation for future longitudinal and intervention research. The innovative aspects of our study are primarily reflected in the following: expanding the research subjects of eHealth literacy and health behaviors from chronic disease patients to a general older adult sample from Shandong Province; being the first to estimate, based on a sample of Chinese older adults, the serial indirect association pattern involving self-efficacy and social support in the association between eHealth literacy and health responsibility; and explaining the indirect role of social support based on traditional Chinese family culture, laying a theoretical foundation for designing intervention programs that are consistent with local cultural characteristics.

## Data Availability

The original contributions presented in the study are included in the article. Further inquiries can be directed to the corresponding author/s.
